# Mediating effects of nurses ‘personal and organizational values between organizational commitment and turnover: Cross-sectional study

**DOI:** 10.1371/journal.pone.0258387

**Published:** 2021-10-14

**Authors:** Wentong Wei, Mengxin Gan, Yanhui Liu, Mengyu Yang, Jingying Liu

**Affiliations:** 1 School of Nursing, Tianjin University of Traditional Chinese Medicine, Tianjin, China; 2 Nursing Department, Tianjin Union Medical Center Nankai University Affiliated Hospital, Tianjin, China; International Medical University, MALAYSIA

## Abstract

**Background:**

The values of individuals and organizations are the core factors driving and guiding nurses’ decision-making and actions. Previous studies mainly focused on the impact of organizational commitment and other influencing factors on turnover intention.

**Aim:**

To explore the mediating effect of personal and organizational values matching the relationship between organizational commitment and turnover intention of nursing staff.

**Methods:**

A cross-sectional survey of 490 subjects in four tertiary hospitals in Tianjin was conducted by convenient sampling. Multivariate regression analysis and structural equation models were used to test each hypothesis.

**Results:**

The results showed that there is a negative correlation between personal and organizational values, organizational commitment and turnover intention, and personal and organizational values played an indirect intermediary role between organizational commitment and turnover intention.

**Conclusions:**

Organizational commitment reduces nurses’ turnover intention indirect through personal and organizational values paths. Leaders can improve nurses’ values as members of the organization, so as to participate in their own work more actively.

**Implications for nursing management:**

Managers should effectively reduce the turnover rate and stabilize the nursing team by improving the organizational commitment and personal and organizational values of clinical nurses.

## Introduction

Nurses’ shortage and intention of losing nurses are the main challenges faced by health care organizations all over the world [1]. In 2020, the total number of registered nurses in China was about 4.45 million, with 3.14 nurses per 1,000 people [2], far lower than the average level of 9 nurses per 1,000 people of the Organization for Economic Cooperation and Development (OECD) [3]. One in five Registered Nurses within 1 year of hire and one-third within 2 years is estimated to leaves the profession [4]. Where major themes of determinants of leaving a position or the profession emerge at the level of the individual, the job and the organization [5]. Turnover may reduce staffing and patient contact time and affect the quality of patient care [6, 7].

Turnover intention refers to “the desire of an individual to leave his or her current job within a certain period” [8]. Turnover intention is an antecedent of leaving, which is considered a key predictor of actual turnover behavior, supported by a great deal of experience and theory [9]. Turnover intention is a cost-effective measure to investigate nurses’ turnover behavior, and numerous studies have employed turnover intention as a measure to predict nurse turnover [10–13]. According to the Price and Mueller causal nurse turnover model, nurse turnover is directly influenced by nurses’ organizational commitment and indirectly through their organizational variables [14]. A large number of domestic and foreign studies [15–18] show that personal organizational fit has a certain influence on employees’ turnover intention and behavior. Research results show that there is a significant negative effect between personal organizational fit and turnover intention [19].

Organizational commitment is a kind of psychological state that describes the relationship between employees and the organization, and it can influence employees’ decisions to continue or terminate their membership [20]. The loss of nurses in medical institutions is a phenomenon that attracts people’s attention in many cases, which is closely related to organizational commitment [21]. A meta-analysis based on 106 primary nurses’ studies found that organizational commitment was one of the strongest indicators of turnover intention [22]. The researchers’ research on turnover focuses on the influence of various potential variables of turnover on turnover intention, and most of the researches show that the higher the organizational commitment of nurses, the lower their turnover intention [23]. As for organizational commitment, the organizational commitment had a negative influence on nurses’ turnover intention [24]. For example, it has been reported that organizational commitment is related to high-quality nursing performance and job retention rates [25, 26]. In contrast, low levels of organizational commitment have been linked to negative work outcomes, such as missed nursing care, poor quality nursing care, decreased organizational citizenship behavior, and high turnover [27, 28]. Kristof [29] found that personal and organizational values have a positive impact on employee satisfaction, organizational commitment, behavior outside the job role, job performance, stress, employee behavior orientation, and turnover rate. Kristof-Brown [30] found that there is a significant correlation between personal and organizational values and organizational commitment. Some researches [31] shows that there is a significant positive correlation between personal and organizational values dimensions and organizational commitment.

Previous studies on organizational commitment and turnover intention mainly focused on the influence of organizational commitment and other influencing factors on turnover intention. In this paper, personal and organizational values are introduced as a mediating variable to discuss the relationship between the three variables.

Person-Organization Fit is a dimension of personal environment fit research, which was first proposed by American psychologist Lewin. He emphasized that all human behaviors are the result of interaction between individuals and their environment, and this fit will bring positive benefits to individuals and organizations [32]. In 1989, when Chatman recorded the concept of personal organizational fit, he put forward that the behavior of members and organizations could influence each other, and the value was the most basic one in all these factors which influence each other [33]. So he believed the individual organization fit referred to the consistent values beyond the individuals and the organizations. Later, more and more scholars have studied the personal and organizational values and found that the compatibility between the individual and the organization could have an impact on the employees’ job performance, organization commitment, and turnover [34–38].

This paper mainly refers to personal and organizational values. Value is a very abstract concept. In psychology, it has cognitive, emotional, behavioral, and other meanings, which is difficult for researchers to understand and grasp. For individuals, values are central factors that drive and guide their decisions and actions; For organizations, values are the core of organizational culture and guide people’s behaviors in organizations [39]. Research shows that personal job matching significantly affects nurses’ turnover intention, and personal and organizational values can adjust the relationship between nurses’ organizational commitment and turnover intention [40]. The research results of a Chinese scholar [41] show that personal and organizational values can directly or indirectly affect employees’ turnover intention.

**Fig 1:**
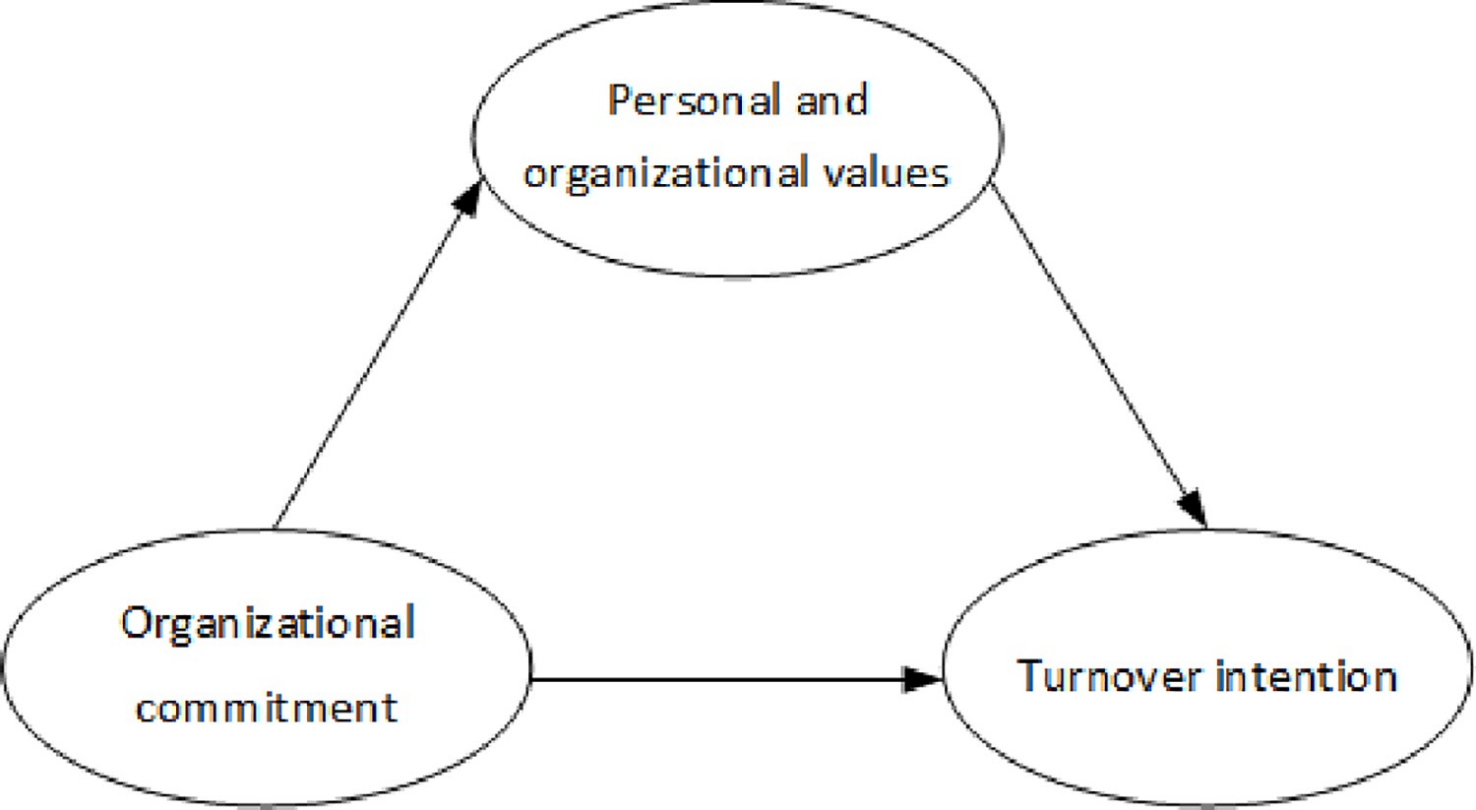
Hypothesized model.

Based on previous research, we proposed the following hypotheses: (a) Organization commitment was adversely related to turnover intention, (b) Person-organization values were adversely related to turnover intention, (c) organizational commitment was positively related to person-organization values (Fig 1, hypothetical model). Nursing managers should pay attention to the personal and organizational values and the impact of organizational commitment when monitoring employee ’turnover intention. Therefore, based on the research on the influence of organizational commitment on turnover intention, this paper introduces the variable of personal and organizational values and focuses on the mediating effect of personal and organizational values on organizational commitment and turnover intention.

## Methods

### Design and participants

A cross-sectional survey of 490 subjects in four tertiary hospitals in Tianjin was conducted by convenient sampling. Inclusion criteria: (1) Registered nurse; (2) At least 1 year of clinical work experience; (3) Voluntary participation. The researchers who distributed the questionnaires explained in advance the purpose of the survey told them that the data was for study use only, that all respondents gave informed consent, and that the questionnaires were filled out anonymously. A total of 490 questionnaires were issued, and 463 valid questionnaires were recovered, with an effective recovery rate of 94.5%. The data collection was completed in April 2019 (See S1 Data).

### Procedure

Before the study, the project was approved by the Medical Ethics Committee of Tianjin University of Traditional Chinese Medicine. The research team explained the purpose and procedure of the study to the director of the nursing department. The research team explained the purpose of the study to the head nurse, and then recruited all nurses to participate in the study. The survey package included questionnaires and an introduction letter about the study’s purposes.

### Survey tool

#### General information questionnaire

General information includes gender, nursing age, educational background, professional title, average monthly income, employment methods.

#### Turnover intention

The scale was compiled by Michael and Spector in 1982 [42] and revised by Li Jingyuan and Li Dongrong for Chinese translation [43], and was divided into three dimensions, including six items. The likert4-level scoring method was adopted: "often" scored 4 points, "occasionally" scored 3 points, "rarely" scored 2 points, and "never" scored 1 point. A total average score of ≤1 indicates a low turnover intention, >1 and ≤2 indicates a low turnover intention, >2 and ≤3 indicate high turnover intention, and >3 indicates high turnover intention. The score indicates the level of turnover intention. The total score of turnover intention is the sum of the scores of six items. DimensionⅠ (items 1 and 6) of turnover intention indicates the possibility of quitting the current job, dimensionⅡ (items 2 and 3) indicates the motivation of finding another job, and dimension Ⅲ (items 4 and 5) indicates the possibility of obtaining an external job. The revised Cronbach’α is 0.773, and the content validity is 67.67%. In our study, the overall reliability of Cronbach’s alpha coefficient of the scale is 0.745.

#### Organizational commitment

The organizational commitment scale was revised by Meyer [44]. A total of 18 projects are divided into three dimensions: emotional commitment, sustained commitment, and normative commitment, with 6 projects in each dimension. Likert’s 5-point scoring method is adopted in the scale, that is, 1–5 means "very inconsistent", "not very consistent", "uncertain", "basically consistent" and "very consistent". To be sure, the higher the score of "quite agree" and "quite agree", the higher the organizational commitment. Cronbach’s alpha coefficient of three dimensions is 0.881, 0.703, and 0.827. In our study, the overall reliability of Cronbach’s alpha coefficient of the scale is 0.804.

#### Person-organization values

Some scholars have used the scales of Resick (2007) to conduct related research [45]. Perceived person-organization value was measured with a 5-item scale. Internal consistency reliability Cronbach’s alpha = 0.94. In 2009, Zhao Huijuan translated and test that the reliability and validity of "person-organization fit" variables meet the measurement requirements [46]. The scale directly measures "value matching", "demand matching" and "ability matching" in a single dimension. Values matching, needs matching and ability matching are independent structures, and the questionnaire measurement has high structural validity. The measurement results were evaluated with the 5-point Likert scale, and employees scored according to their personal feelings. One representative is very inconsistent and five representatives are very consistent. The reliability coefficient of the scale in this study was 0.826.

### Statistical analysis

The data were analyzed by SPSS 22.0 and AMOS. General characteristics are described by descriptive statistics. Pearson correlation analysis was used to evaluate the relationships among all subscales. Structural equation modeling was conducted to verify the relationships among personal and organizational values, organizational commitment, and turnover intention. The mediating effects of personal and organizational values were also tested.

### Ethical considerations

The researchers declare in advance that the questionnaire data would only be used for research, and the participants would voluntarily sign an informed consent form and fill it in anonymously.

## Results

### Participant characteristics and work-related data

The study participants were 463 registered nurses, mostly female (98.3%), ranging in age from 22 to 55 years old, with an average age of (M = 34.79, SD = 8.413). Most of the respondents have been nursing for more than 6 years (73.2), 64.1% have a bachelor’s degree or above. Other demographic characteristics were included in Table 1.

**Table 1:** Sociodemographic characteristics of nurses (N = 463).

Variables	N	%	Turnover intention		P
			Mean	SD	
**Gender**					0.00**
Female	455	98.30	14.89	3.71	
Male	8	1.70	20.13	1.89	
**Age**	34.85±0.39				0.04*
**Working Years**					0.43
Less than three years	77	16.60	15.16	3.47	
Three to five years	47	10.20	15.21	3.81	
Six to ten years	93	20.10	15.47	4.00	
Eleven to fifteen years	96	20.70	14.90	3.79	
Over fifteen years	150	32.40	14.57	3.68	
**Marital Status**					0.38
Unmarried	124	26.80	15.10	3.84	
Married	328	70.80	14.96	3.72	
Divorced	8	1.70	15.38	3.20	
Widowed	3	0.60	11.33	4.93	
**Official academic credentials**					0.03*
Technical secondary school	13	2.80	12.92	4.54	
Junior college	153	33.10	14.56	3.73	
Regular college course	295	63.70	15.27	3.68	
Master’s and above	2	0.40	18.00	4.24	
**The title of a technical post**					0.41
Nurse	108	23.30	15.38	3.50	
Senior nurse	166	35.90	15.06	3.94	
Nurse-in-charge	179	38.70	14.73	3.78	
Associate chief nurse and above	10	2.10	13.90	2.02	
**Average monthly earnings**					0.07
Under ¥3000	24	5.20	14.92	3.34	
¥3000–5000	204	44.10	15.44	3.47	
¥5000–8000	227	49.00	14.66	4.02	
Over ¥8000	8	1.70	12.88	2.10	
**Employment way**					0.16
Officially enrolled	345	74.50	14.87	3.79	
Personnel Agency	41	8.90	15.44	3.54	
Contract system	19	4.10	13.79	3.84	
Dispatching system	58	12.50	15.74	3.53	

**p < 0.01

*p < 0.05.

### Means, standard deviations, and correlations among variables

Mean scores, standard deviations and correlations among scales are shown in Table 2. All scales were correlated significantly with each other (p< 0.01). There was a positive correlation between organizational commitment and personal and organizational values. Organizational commitment as well as between personal and organizational values was negatively correlated with turnover intention.

**Table 2:** Means, standard deviation (SD), and correlations of turnover intention, organizational commitment, and personal and organizational values.

	Mean	SD	1	2	3	4	5	6	7	8	9
1.Personal and organizational values	18.59	3.37	1								
2. Turnover intention	14.98	3.75	-0.64**	1							
3. Organizational commitment	66.00	10.72	0.72**	-0.72**	1						
4. Turnover intention (Quit the existing job maybe)	4.91	1.78	-0.52**	0.90**	-0.62**	1					
5. Turnover intention (Looking for other job possibilities)	4.56	1.58	-0.54**	0.85**	-0.60**	0.68**	1				
6. Turnover intention (The possibility of getting other jobs)	5.48	1.14	-0.48**	0.66**	-0.53**	0.45**	0.35**	1			
7. Organizational commitment (Emotional commitment)	23.12	3.96	0.59**	-0.62**	0.83**	-0.53**	-0.52**	-0.42**	1		
8. Organizational commitment (Continuous commitment)	20.97	4.07	0.54**	-0.54**	0.76**	-0.45**	-0.45**	-0.43**	0.42**	1	
9. Organizational commitment (Specification commitment)	21.80	4.54	0.63**	-0.66**	0.90**	-0.57**	-0.56**	-0.46**	0.74**	0.56**	1

**p < 0.01.

### Multivariable linear regression

Taking the turnover intention as the dependent variable and two statistically significant variables (organizational commitment, personal and organizational values) as independent variables, multiple linear regression was conducted. The results showed that organizational commitment and personal and organizational values were the main influencing factors for turnover intention. It explained 54.9% of the total variation (Table 3).

**Table 3:** The results of multivariable linear regression to predict variables related to turnover intention.

Variable	β	SE	β’	t	VIF	P
constant	32.645	0.754		43.321		**
organizational commitment	-0.192	0.016	-0.550	-12.235	2.069	**
personal and organizational values	-0.267	0.050	-0.240	-5.341	2.069	**

R^2^ = 0.550, Adjusted R^2^ = 0.549, F = 281.634

** p < 0.01.

### Structural equation modeling results

**Fig 2:**
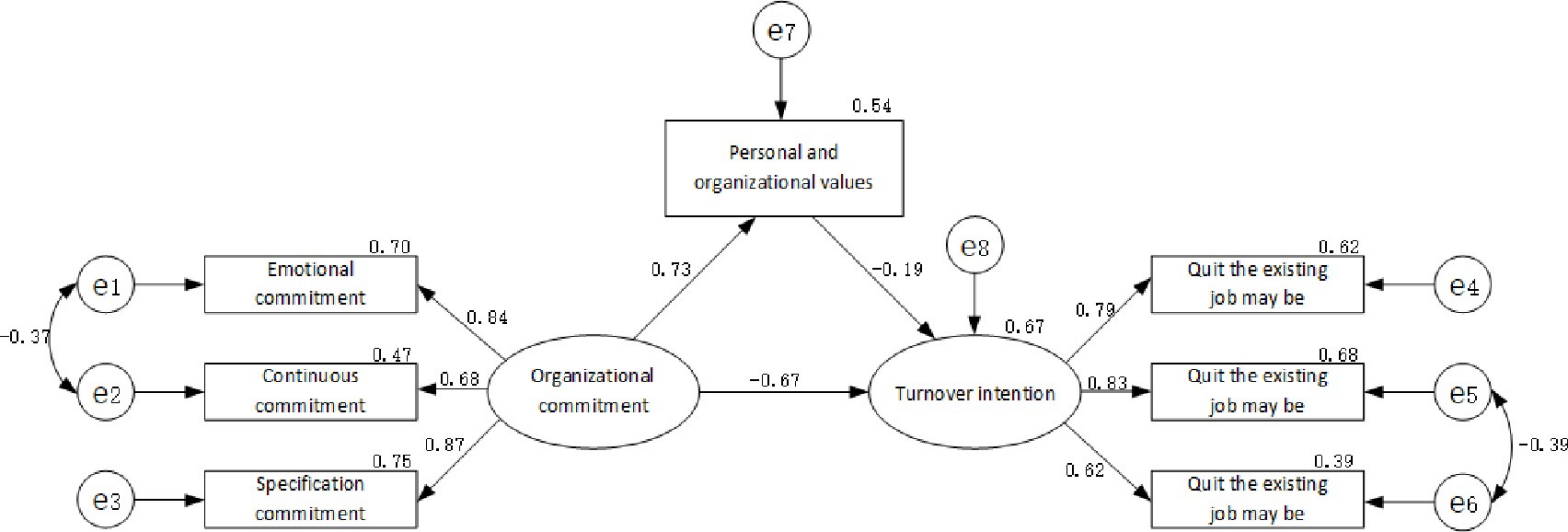
Structural equation modeling results.

A model of the relationship among personal and organizational values, organizational commitment, and turnover intention (Fig 2). After correcting the residual based on modification indices, the model fit indices indicated that the measurement model was suitable: χ2/df = 2.547, RMSEA = 0.058, GFI = 0.984, CFI = 0.990, AGFI = 0.956 and TLI = 0.980 (Table 4).

**Table 4:** Structural equation modeling fit indices.

	χ2	df	χ2/df	RMSEA	NFI	CFI	GFI	AGFI	TLI
Mediation model	25.467	10	2.547	0.058	0.984	0.990	0.984	0.956	0.980

Abbreviations: AGFI, adjusted goodness of fit index; CFI, comparative fit index; df, degree of freedom; GFI, the goodness of fit index; NFI, normed fit index; RMSEA, root mean square error of approximation; TLI, Tucker‐Lewis fit index.

Bootstrap analysis method was adopted, and repeated sampling was conducted for 2000 times to check whether the mediation effect was significant. The confidence interval was set at 95%, and the results are shown in Table 5. The total indirect effect is -0.050, indicating that personal and organizational values play a partial mediating role between organizational commitment and turnover intention, and the indirect effect accounts for 17.30% of the total effect, that is, 17.30% of the effect of organizational commitment on turnover intention is through personal and organizational values.

**Table 5:** Direct and indirect effects for the final model.

Model pathways	β	S.E.	95%CI.	Effect Ratio(%)	p
**Direct Effects**	-0.239	0.027	[-0.527,-0.799]	82.70	**
**Indirect Effects**	-0.050	0.019	[-0.040,-0.245]	17.30	**
**Total Effects**	-0.289	0.017	[-0.736,-0.865]	100.00	**

**p < 0.01.

## Discussion

The purpose of this study is to explore the relationship between personal and organizational values, organizational commitment, and turnover intention. Our research found that the personal and organizational values, organizational commitment, and turnover intention were negatively correlated, and the personal and organizational values and organizational values played a mediating role between organizational commitment and turnover intention.

### Differences in demographic variables and turnover intention

Researchers found that there are significant differences in the scores of nurses’ turnover intention between different genders (p < 0.01). Male nurses score higher on turnover intention, which may be influenced by traditional concepts and Chinese social culture, and is not objective to their career development prospects [47, 48]. Most male nurses do not choose nursing specialty voluntarily, which is mostly caused by adjustment. They don’t have a complete understanding of nursing profession, lack correct understanding of nursing profession, have no good sense of professional identity, and have a high level of turnover intention [49].

This research is that nurses of different ages have significant differences in their willingness to leave (p < 0.05). Compared with older nurses, younger nurses have higher turnover intention, which is due to their lower problem-solving ability and lack of work skills. Our finding was similar to that of previous research [50, 51].

This study shows that nurses with higher educational background have higher turnover intention (p < 0.05).With the development of higher nursing education, there are more and more nursing backbones with bachelor degrees or above. They have higher professional development needs, and hope to be recognized and respect. Nurses with bachelor degrees or above, in particular, have higher expectations for their professional development [52, 53] (See Table 1).

### Correlation among nurses’ organizational commitment, personal organizational values and turnover intention

A negative correlation was found between organizational commitment and turnover intention (r = -0.72, p < 0.01) and a negative correlation between personal and organizational values and turnover intention (r = -0.64, p < 0.01). The average score of the turnover intention of participants was 14.98 (3.75) (See Table 2). The dimension with the highest score ranking is the possibility of getting another job. These results show that nurses’ turnover intention is at a higher level. In this study, nurses’ turnover intention is slightly lower than that of a study conducted by Liu et al [54], which a total of 291 of the 1216 nurses (23.9%) with a higher turnover intention tended to want to leave more strongly (mean score of turnover intention >3). This difference may be due to the improvement of nurses’ status in China and the application of standardized clinical nursing procedures in hospitals, resulting in a slight decline in nurses’ turnover intention.

Organizational commitment was a significant predictive factor in nurse turnover intention. The results of this study are consistent with previous studies, some scholars suggest that most nurses working in emergency had low affective and normative commitment which have a negative significant influence on turnover intentions. Low affective commitment means that nurses have a low perception to be part of the hospital organization. They have a low sense of belonging and not happy to be part of the nursing team in these hospitals [44, 55–57].

From the multi-variable linear regression model conducted to predict variables related to turnover intention, we can see that higher level of organizational commitment and personal-organizational values were predicted lower turnover intention. When nurses think that hospitals and departments must pay attention to his or her contributions, care about their interests and career development, this will reduce their turnover intention, and lead to a higher organizational commitment and personal and organizational values [54, 58].

Combined with Pearson correlation analysis results that organizational commitment and personal and organizational values can reduce turnover intention rates [59, 60]. The more employees care about their organization, the more likely they are to stay. Therefore, this may improve their values as members of the organization, leading to more active involvement in their work [61]. Other studies have confirmed that personal and organizational values have a positive impact on employees’ organizational commitment [62]. At the same time, they will become more dependent on the organization and think less about leaving it. First, in the organization’s personnel recruitment and selection process, employees with higher compatibility between personal values and organizational values should be selected as much as possible [63].

### Mediating role of personal organizational values in organizational commitment and turnover intention

The analysis of the results of this research path shows that nurses’ organizational commitment and personal organizational values have an impact on turnover intention, among which organizational commitment has a direct impact of -0.239, the indirect impact of -0.050, the total impact of -0.289, and the indirect impact of 17.30% (See Table 5).

Our research results are consistent with Tan [39] shows that personal and organizational values play a partial intermediary role between organizational commitment and turnover intention. To fully understand the influence of organizational commitment on turnover intention, the mediating effect of personal and organizational values can be considered. In organizations with low matching values between individuals and organizations, nurses are weak in team consciousness and lack the enthusiasm to achieve high performance. When nursing staff feels higher organizational commitment, if their values can match the organizational values, they will consistent with organizational actions to improve organizational performance [64, 65].

The results show that organizational commitment, personal and organizational values are highly correlated with turnover intention. Considering the unique characteristics of the medical system, policies, and cultural context, it was essential to conduct this study in China. To comprehensively prevent and reduce nurse turnover intention, the mediating effect of personal and organizational values on organizational commitment and turnover intention. The model pathway is as follows: [organizational commitment →personal and organizational values], [personal and organizational values → turnover intention], and [organizational commitment → turnover intention]. From the results, we can see that all the paths were significant (p < 0.01) (See Fig 2). Therefore, in nursing work, hospital leaders should guide employees to find the consistency between personal needs and organizational needs, and help them find a sense of belonging to the hospital [66, 67].

This study explores the mediating effect of personal and organizational values between organizational commitment and turnover intention. In organizations with a low matching degree of personal and organizational values, employees are generally not enthusiastic about various things, and their team consciousness is not strong [63, 68]. When the values of individuals and organizations are highly matched, employees can quickly adapt to the organization’s work rhythm, better grasp the work requirements, and greatly improve work efficiency. Our research also shows that the value matching between individuals and organizations has a great influence on emotional commitment and Specification commitment, while continuous commitment reflects the negative tendency of employees to leave the enterprise.

Therefore, managers should help employees correct their work attitudes and behaviors [69, 70], encourage employees to reflect their work values, improve the matching degree between employees and the organization ’values, and make employees feel that organization give a more emotional commitment [71], overcome various difficulties, succeed in their work, and prefer to work in the organization [13, 72]. Some mediating effects we found indicate that there may be other mechanisms to explain the impact of organizational commitment on turnover intention. Therefore, future research should explore the multiple intermediary mechanisms of organizational commitment affecting turnover intention.

### Limitations and future research directions

There are certain limitations to this paper. We preferred to use survey rather than sampling, which is one of the limitation of our research. First of all, in the relevant literature, there is little research on the relationship between personal and organizational values, organizational commitment, and turnover intention. Secondly, we emphasized some intermediary role in the survey results, but do not consider the influence of demographics. Thirdly, the research was only conducted in Tianjin, China, and the results were not extensive.

To make an in-depth study of the factors affecting turnover intention, we can expand the sample size, expand the scope, change cities, or conduct further research in different cities by examining various variables. Besides, considering the cross-sectional nature of the data, we should adopt a different method, and choose a longitudinal research method in the future, and use a random survey or monitoring method to track the results at any time and for a long time.

## Conclusion

To sum up, we studied the relationship between organizational commitment, personal and organizational values, and turnover intention. It is found that personal and organizational values play an intermediary role between the organization’s commitment to employees and employees’ willingness to leave the organization. According to these results, it is necessary to formulate and implement intervention strategies to improve nurses’ organizational commitment and personal and organizational values, which is the key to reducing their intention to leave.

We hope that the hospital can formulate corresponding policies, such as raising the annual salary of nurses, expanding the enrollment scale of nursing staff, and arranging reasonable vacation time. By organizing the training of head nurses, we can improve the leadership of department management, and make nurses have confidence in their career prospects, and work with a positive attitude. At the same time, nursing managers can reduce the turnover intention of clinical nurses and improve the shortage of nursing staff by strengthening the matching of employees and organizational values and organizational commitment.

## Implications for nursing management

Administrators should effectively reduce employee turnover rate and stabilize the nursing team by improving the organizational commitment and personal and organizational values of clinical nurses.

## Supporting information

S1 Data (SAV)
